# Air Pollution, Airway Inflammation, and Lung Function in a Cohort Study of Mexico City Schoolchildren

**DOI:** 10.1289/ehp.10926

**Published:** 2008-02-08

**Authors:** Albino Barraza-Villarreal, Jordi Sunyer, Leticia Hernandez-Cadena, Maria Consuelo Escamilla-Nuñez, Juan Jose Sienra-Monge, Matiana Ramírez-Aguilar, Marlene Cortez-Lugo, Fernando Holguin, David Diaz-Sánchez, Anna Carin Olin, Isabelle Romieu

**Affiliations:** 1 Instituto Nacional de Salud Pública, Cuernavaca, México; 2 Environmental Epidemiological Research Centre (CREAL), IMIM, Barcelona, Spain; 3 Hospital Infantil de México, Federico Gómez, Mexico, D.F., Mexico; 4 Comisión Federal de Protección contra Riesgos Sanitarios (COFEPRIS), México, D.F. Mexico; 5 Department of Pulmonary Allergy and Critical Care, Emory University School of Medicine, Atlanta, Georgia, USA; 6 Human Studies Division, U.S. Environmental Protection Agency, Chapel Hill, North Carolina, USA; 7 Department of Occupational and Environmental Medicine, Sahlgrenska University Hospital, Gothenburg, Sweden

**Keywords:** air pollution, airway inflammation, asthma, epidemiology, lung function, schoolchildren

## Abstract

**Background:**

The biological mechanisms involved in inflammatory response to air pollution are not clearly understood.

**Objective:**

In this study we assessed the association of short-term air pollutant exposure with inflammatory markers and lung function.

**Methods:**

We studied a cohort of 158 asthmatic and 50 nonasthmatic school-age children, followed an average of 22 weeks. We conducted spirometric tests, measurements of fractional exhaled nitric oxide (Fe_NO_), interleukin-8 (IL-8) in nasal lavage, and pH of exhaled breath condensate every 15 days during follow-up. Data were analyzed using linear mixed-effects models.

**Results:**

An increase of 17.5 μg/m^3^ in the 8-hr moving average of PM_2.5_ levels (interquartile range) was associated with a 1.08-ppb increase in Fe_NO_ [95% confidence interval (CI), 1.01–1.16] and a 1.07-pg/mL increase in IL-8 (95% CI 0.98–1.19) in asthmatic children and a 1.16 pg/ml increase in IL-8 (95% CI, 1.00–1.36) in nonasthmatic children. The 5-day accumulated average of exposure to particulate matter < 2.5 μm in aerodynamic diamter (PM_2.5_) was significantly inversely associated with forced expiratory volume in 1 sec (FEV_1_) (*p* = 0.048) and forced vital capacity (FVC) (*p* = 0.012) in asthmatic children and with FVC (*p* = 0.021) in nonasthmatic children. Fe_NO_ and FEV_1_ were inversely associated (*p* = 0.005) in asthmatic children.

**Conclusions:**

Exposure to PM_2.5_ resulted in acute airway inflammation and decrease in lung function in both asthmatic and nonasthmatic children.

Exposure to air pollution has been associated with decrements in lung function (Castillejos et al. 1995; Gauderman et al. 2007; Moshammer et al. 2006; Romieu et al. 1997, 1998; Svendsen et al. 2007) and an increase in respiratory symptoms (Castillejos et al. 1992; Leonardi et al. 2000), effects to which asthmatic children appear more susceptible (Romieu et al. 1996; Ward and Ayres 2004). Experimental and epidemiologic studies have also shown that fossil fuel–combustion products act as an adjuvant in the immune system and may lead to enhancement of allergic reaction (Nel et al. 1998) to airway inflammation in susceptible subjects (Holguin et al. 2007; Leonardi et al. 2000; Yeatts et al. 2007) with potential long-term effects (Calderon-Garcidueñas et al. 2006). However, few longitudinal epidemiologic studies have evaluated the impact of fossil fuel combustion on airway inflammatory response (Fischer et al. 2002; Koenig et al. 2003, 2005; Steerenberg et al. 2001) using an integrated approach including markers of airway inflammatory response and lung function changes.

Asthma is a complex disease characterized by inflammation and hyperresponsiveness of the airways. Nitric oxide signaling pathways have been implicated in the regulation of airway hyperresponsiveness and recently of fractional exhaled nitric oxide (Fe_NO_) (Van Amsterdam et al. 2000) and the pH of exhaled breath condensate (EBC) samples has been proposed as a noninvasive biomarker to assess airway inflammation (Rosias et al. 2004). Exhaled NO is produced endogenously in the airways from l-arginine by NO synthase (NOS) (Silkoff et al. 2000), and two constitutive isoforms and cytokine-induced overexpression of NOS are likely to contribute to its increase (Wu et al. 2007). Similarly, increased cytokine levels have been described in asthmatic patients and interleukin (IL)-8 may contribute to inflammatory response in the airways (Narayanan et al. 1999).

The aim of the present study was to evaluate the association of air pollutant and traffic-related exposures with Fe_NO_ levels, IL-8 levels in nasal lavage, pH of EBC, and lung function in a cohort of school-age asthmatic and nonasthmatic children. We hypothesized that short-term exposure to air pollution and traffic-related emissions was associated with increased airway inflammation and decreased lung function.

## Material and Methods

### Study design

A dynamic panel study of asthmatic and nonasthmatic schoolchildren living in Mexico City was conducted during June 2003–June 2005. The children were enrolled during the first 10 months of the study (June 2003–April 2004) and followed for an average of 22 weeks. Spirometric tests and measurements of Fe_NO_ levels, IL-8 levels in nasal lavage, and pH of EBC were conducted during the follow-up.

### Location and population

The study population consisted of children living in three Mexico City municipalities, Iztapalapa, Iztacalco, and Netzahualcóyotl, where high levels of traffic-related emissions are the major source of pollutants. The Mexican Ministry of Transport and Communications (Secretaría de Transportes y Vialidad, Mexico City, Mexico) reports that on weekdays about 35,000 vehicles pass through the intersection of two highways in one direction, and that about 2,000 diesel-fueled vehicles per day use one of the highways in the study area (Gobierno del Distrito Federal 1999). Similarly, the traffic count conducted in this area indicated a flow of between 303 and 1,840 vehicles per hour (Romieu et al. 2008a).

**Figure 1:**
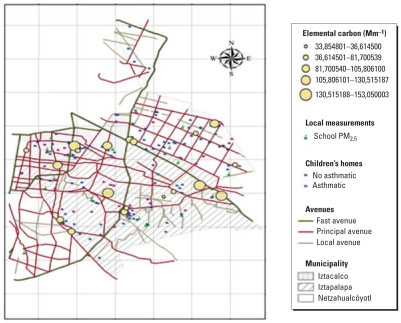
Map of the study area, illustrating the location of the major roads and schools and the homes of the asthmatic and nonasthmatic children in each municipality, Mexico City, 2003–2005.

One hundred fifty-eight asthmatic children attending the Hospital Infantil de Mexico Federico Gómez, one of the largest pediatric hospitals in the city (Mexico City), were invited to participate in the study. The diagnosis and severity of their asthma were based on clinical symptoms and response to treatment and rated by a pediatric allergist as mild (intermittent or persistent), moderate, or severe according to the Global Initiative for Asthma (GINA) guidelines (Global Initiative for Asthma 2006). Fifty nonasthmatic children were recruited by asking the asthmatic children to invite a schoolmate or a friend from their neighborhood. The children in both groups were between 6 and 14 years of age. They lived in the study area, attended public schools located close to their home, were volunteers, and were not selected using probability-based sampling. Figure 1 presents the study area, showing schools, monitoring stations, major highways, and the location of the children’s homes. All procedures were explained to the parents, who signed an informed consent form. The children also gave their informed assent. The study protocol was reviewed and approved by ethics committees at both the National Institute of Public Health and the Hospital Infantil de Mexico.

### Collection of health outcomes

We collected data using a general-purpose questionnaire (adapted from existing survey instruments) on sociodemographic variables, past health history, and potential indoor environmental exposures (tobacco smoke and pets in the home). Information on allergy test results, medication, and medical visits in the preceding 2 years was obtained from the medical record. At baseline and every 15 days during follow-up, a respiratory symptoms questionnaire was applied, anthropometric measurements were taken, spirometric tests performed, Fe_NO_ levels measured, and samples of nasal lavage and EBC obtained. Spirometric tests were performed on 158 asthmatic children (1,503 measurements) and 50 nonasthmatic children (591 measurements). Measurements were repeated an average of 11 times (range, 5–21) per subject during the study period. We were unable to take all samples for inflammatory markers from all the children, for logistic reasons. We obtained 702 measurements of Fe_NO_ from 126 asthmatic children and 302 from 50 nonasthmatic children, 759 measurements of IL-8 in nasal lavage from 129 asthmatic children and 285 from 45 nonasthmatic children, and 551 measurements of pH in exhaled breath condensate from 119 asthmatic children and 201 from 44 nonasthmatic children.

### Spirometry

The spirometric tests were performed according to American Thoracic Society (ATS) specifications (ATS 1995) using an EasyOne spirometer (ndd Medical Technologies, Andover, MA, USA). The tests were conducted in a room with stable temperature and relative humidity. All lung function tests were performed by the same technicians, and the best of three technically acceptable tests was selected.

### Exhaled nitric oxide determination

The levels of Fe_NO_ were measured following the ATS guidelines (ATS 1999) during out-patient visits to a clinic. Children were seated for at least 5 min before commencing the measurement and throughout the procedure; all measurements were conducted indoors to minimize inhaled NO-free external air. NO was measured by chemiluminescence, using a continuous analyzer (CDL 88 sq; ECO Physics, Ann Arbor, MI, USA). The Fe_NO_ reading was displayed on the monitoring system and the mean of three acceptable tests was taken.

### Nasal lavage

Nasal lavage was performed following the methodology proposed by Diaz-Sanchez et al. (1994), with the subject sitting with the nasopharynx closed while tilting the neck back 45° from the vertical. Five milliliters of warm (37°C) normal saline is instilled into each nostril by pipette. After 10 sec, during which the subject shakes the head softly from side to side, the subject brings the head forward, expelling the wash fluid into a plastic receptacle. The subject then performs up to four further nasal washes at 30-sec intervals, with each wash being collected in a separate tube. We measured different cytokines including IL-8, interferon gamma, IL-6, and IL-10 levels in nasal lavage in the laboratory of D. Diaz–Sanchez, using commercially available ELISA kits according to the manufacturer’s instructions. However, except for IL-8, the levels in most of the samples were below the detection limit and we report only the IL-8 results. For logistic reasons, we did not determine cellular composition.

### EBC collection

EBC was collected using an R-tube, and the breath was cooled by placing an aluminum cooling sleeve over the disposable polypropylene tube (Hunt 2002). Samples were obtained following the ATS/ERS (European Respiratory Society) Task Force recommendations (Horvath et al. 2005; Hunt 2002). Participants were asked to breathe tidally through the mouthpiece connected to the R-tube for 10 min to collect approximately 2 mL of exhaled breath fluid, which was aliquoted and frozen to −70°C within 15 min of collection.

### Exposure assessment

Exposure was estimated from outdoor PM_2.5_ (particulate matter < 2.5 μm in aerodynamic diameter), NO_2_ and O_3_ concentrations recorded by the Mexico City government at four fixed-site central monitoring [Red Automática de Monitoreo Afmosférico (RAMA)] locations within the study area (Cerro de la Estrella and Hangares; Merced; Universidad Autonoma Metropolitana, Iztapalapa; and La Perla). Daily average, maximum moving average and 8-hr maximum ozone, nitrogen dioxide, and PM_2.5_ concentrations, and meteorologic data (temperature and humidity) were obtained for all (505) days of the study period. The home of each participating child was georeferenced using a geographic information system (GIS), and the closest monitoring station was assigned to the child. All children attended public schools located close to their home, and no fixed-site monitoring station was > 5 km from a child’s home or school. We also conducted monitoring at each school for three 15-day periods during the follow-up to validate data obtained from the fixed-site monitoring stations (RAMA). Local daily 24-hr average PM_2.5_ was determined using Mini-Vol portable air samplers (version 4.2; Airmetrics, Eugene, Oregon, USA) with 47-mm Teflon filters (R2PJ047; Pall Gelman, Ann Arbor, MI, USA) and flows set at 5 L/min, and 7-day integrated data for NO_2_ and O_3_ concentrations were obtained using Ogawa passive samplers (Ogawa USA, Pompano Beach, FL, USA) (Watson et al. 1999). Samplers were located outside the 37 schools, generally on the roof, at a height of up to 4 m and far from any objects (e.g., trees, buildings) that would prevent air flow. Gravimetric analysis of the 47-mm Teflon filters was performed at the air laboratory of the National Center for Environmental Research and Training (CENICA) in Mexico City. The NO_2_ and O_3_ filters were assembled at the Mexico City laboratory. After exposure, all badges were placed in sealed bags and sent to the Harvard School of Public Health for chemical analysis (Levy et al. 1998).

### Statistical analysis

The basic characteristics of the two groups of children were compared by bivariate analysis using the *t*-test, the Fisher exact test, or the chi-square test, depending on variable type. The short-term association of traffic-related pollutants (PM_2.5_, O_3_, and NO_2_ concentrations at the fixed-site monitoring stations) with the health outcomes was studied using linear mixed-effects models, considering models for continuous and binary response. This enabled us to appreciate the variability within and between subjects. We ran models with both random intercept and random slope, and with random intercept only. Because the coefficients were similar in both types of model, we present results from the linear mixed model with random intercept only. The model is as follows:


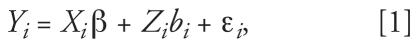


where *X_i_* = *Z_i_X_i_* is the appropriate (*n_i_x p*) matrix of known covariates with fixed effects β and subject-specific effects *b_i_* and ε*_i_* in an *n_i_*-dimentional vector of residual components. Another advantage of the models used is that they do not discard subjects with incomplete data. We determined the goodness of fit of each model using residual diagnosis and the Hausman specification test (Hausman 1978).

Based on our previous work and international studies conducted among asthmatic children (Delfino et al. 2004, 2006; Romieu et al. 1996, 2002, 2004; Schildcrout et al. 2006), we hypothesized *a priori* that the acute effect of air pollutants on pulmonary functions would occur with lags of 1–2 days and that a larger effect would be observed when considering cumulative exposure over several (up to 5) days, whereas for inflammatory markers (Fe_NO_ levels, IL-8 levels, pH of EBC) we hypothesized a shorter response with 0- to 1-day lag. We modeled several pollution exposure indices (8-hr maximum moving average, 24-hr average, 24-hr maximum average, accumulated days). Pearson correlations were determined between air pollutant levels and various climatic variables. Fe_NO_ and IL-8 levels were not normally distributed and were log transformed. Models were adjusted for potential confounding factors including sex, body mass index, previous day minimum temperature, corticoid use, and chronological time. Other variables such as age, socioeconomic index (considering mother education and school type), outdoor activities, atopic status, exposure to environmental tobacco smoke, use of antiallergy medicine, and season were not significant (*p* > 0.10) and did not alter the results by >1%. Analyses were conducted using STATA (version 9.2; StataCorp., College Station, TX, USA).

## Results

Table 1 presents the characteristics of the study population. The median age of participants was 9.6 years [quartile (Q) 25: 7.9; Q75: 11.0) for the asthmatic and 9.3 (Q25: 7.9; Q75: 11.5) for the nonasthmatic children. Of the asthmatic children, 55% were classified as having mild intermittent, 26.9 % as having mild persistent, and 17.5% as having moderate persistent asthma, according to the GINA guidelines. Of the asthmatic children, 6% used an inhaled corticosteroid and 10% were prescribed antibiotics on at least one occasion during follow-up. Eighty-nine percent of the asthmatic children and 72% of the non-asthmatic children had positive skin prick tests. The most common sensitivities were to house dust mite (*Dermatophagoides pteronyssinus*), cat (Fel d 1), and cockroach (*Blatella americana*) allergens*.*

Participants with asthma had higher average levels of Fe_NO_ (*p* = 0.001) and lower average levels of IL-8 (*p* = 0.002). The pH measurements in EBC were similar in both groups (*p* = 0.632).

### Environmental exposure data

**Figure 2:**
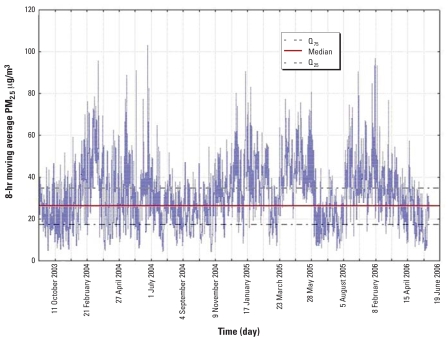
Eight-hour moving average concentrations of PM_2.5_ (μg/m^3^) during the study period, Mexico City, 2003–2005.

The 8-hr moving average PM_2.5_ ranged from 4.24 to 102.8 μg/m^3^ during the study period, with a mean of 28.9 μg/m^3^. It exceeded 30 μg/m^3^ on 52% of the days (Figure 2). The 8-hr moving average NO_2_ levels ranged from 14.9 to 77.6 ppb with a mean of 37.4 ppb. The 8-hr moving average O_3_ level ranged from 4.9 to 86.3 ppb with a mean of 31.6 ppb (Table 2). The correlation between PM_2.5_ and O_3_ was *r* = 0.46 (*p* = 0.000). The correlations between O_3_ and NO_2_ and NO_2_ and PM_2.5_ were *r* = 0.28 (*p* = 0.000) and *r* = 0.61 (*p* = 0.000) respectively. Local measurements conducted at the children’s schools were correlated with levels at the central monitoring stations (*r* = 0.77 for PM_2.5_, *r* = 0.21 for NO_2_, and *r* = 0.60 for O_3_). The mean (± SD) of local measurements was 26.3 ± 12.5 μg/m^3^ for PM_2.5_, 35.05 ± 12.6 ppb for NO_2_, and 26.9 ± 9.5 ppb for O_3_.

### Association between health outcomes and air pollutants

The associations between traffic-related pollutants and main outcomes are shown in Table 3 for asthmatic children and Table 4 for nonasthmatic children. After adjusting for sex, body mass index, corticoid use, previous day minimum temperature, and chronological time, an increase of 17.5 μg/m^3^ in the 8-hr moving average of PM_2.5_ levels [equivalent to one interquartile range (IQR)] was associated with a 1.08-ppb increase in Fe_NO_ [95% confidence interval (CI), 1.01–1.16] in asthmatic children, whereas in nonasthmatic children the association was marginally significant (*p* < 0.081). In contrast, an increase of 17.5 μg/m^3^ in 8-hr moving average PM_2.5_ levels (IQR) was more strongly associated with IL-8 levels in nonasthmatic children (1.16 pg/mL increase; 95% CI, 1.00–1.36) than in asthmatic children (1.08 pg/mL increase; 95% CI, 0.98–1.19). Similarly, the 8-hr moving average of NO_2_ was associated with an increase in Fe_NO_ in asthmatic children but not in nonasthmatic children, whereas it was associated with IL-8 in nonasthmatic children but not in asthmatic children. The 8-hr moving average of O_3_ was associated with both Fe_NO_ and IL-8 in asthmatic children. The pH of EBC was significantly associated with O_3_ exposure only in asthmatic children. Further adjustment for the use of allergic medicine did not modify these results.

**Figure 3:**
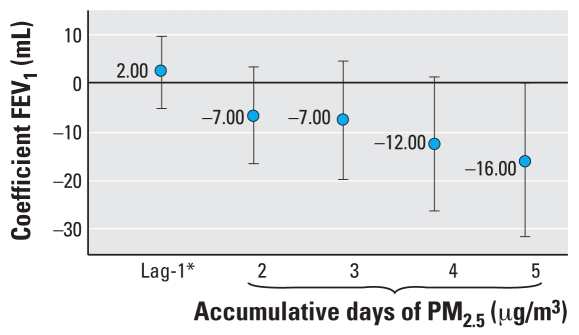
Association between FEV_1_ and PM_2.5_ average cumulative exposure in asthmatic children living in Mexico City, 2003–2005. *Twenty-four-hour maximum.

Cumulative PM_2.5_ exposure over the 5 preceding days was associated with a significant decrement in forced vital capacity (FVC) in both asthmatic and nonasthmatic children, whereas the decrement in FEV_1_ was significant only in asthmatic children (Figure 3). For FEV_25–75_ (the middle 25–75% of the forced expiratory volume measurement), the associations were not significant (Tables 3 and 4). Various models were run considering different lags; however, the effect of PM_2.5_ on FVC and FEV_1_ (FEV in 1 sec) was stronger for a 1-day and a 5-day lag (data not shown). No significant associations between NO_2_ and O_3_ and lung function tests were observed in asthmatic or nonasthmatic children.

Among asthmatic children, the increase in Fe_NO_ was associated with a decrease in FEV_1_ (*p* = 0.005), particularly among those with mild intermittent and moderate asthma. IL-8 was also inversely associated with FEV_1_ but the association did not reach significance (*p* = 0.18).

Respiratory symptoms were monitored during follow-up. Cough and wheezing were associated with same-day exposure to air pollutants. An increase in PM_2.5_ of 17.5 μg/m^3^ was associated with an 11% increase in cough [odds ratio (OR) = 1.11; 95% CI, 106–1.17) and a 6% increase in wheezing (OR = 1.06; 95% CI, 0.99–1.13). An increase in NO_2_ of 34 ppb (1-hr maximum) was associated with a 10% increase in cough (OR = 1.10; 95% CI, 1.04–1.16) and a 10% increase in wheezing 10% (OR = 1.10; 95% CI, 1.03–1.18). And an increase in O_3_ of 48 ppb (1-hr maximum) with a 9% in cough (OR = 1.09; 95% CI, 1.03–1.15) but no significant increase in wheezing. Among nonasthmatic children, only an increase in cough related to cumulative NO_2_ exposure (OR = 1.22; 95% CI, 1.03–1.45 for an increase of 24.5 ppb in 2-day cumulative exposure) was observed.

In multipollutant models including O_3_ as well as PM_2.5_, we noted that among asthmatic children O_3_ remained significantly related to inflammatory markers (Fe_NO_, IL-8, and pH of EBC) whereas PM_2.5_ remained inversely related to pulmonary functions (FEV_1_ and FVC) but lost its significant effect on Fe_NO_. Among nonasthmatic children, the inverse relation between PM_2.5_ and FEV_1_ and FVC persisted, whereas PM_2.5_ and O_3_ had a lesser effect on inflammatory markers (data not shown).

A large proportion (72%) of the nonasthmatic children were atopic, so we repeated the analysis including only atopic children without asthma. In this subgroup we observed a significant increase of Fe_NO_ related to PM_2.5_ exposure, suggesting that atopy increases the Fe_NO_ response (data not shown).

## Discussion

The results of the present cohort study of asthmatic and nonasthmatic children show that Fe_NO_ levels, IL-8 levels in nasal lavage, pH of EBC, and changes in lung function are associated with acute exposure to traffic-related air pollutants. Changes in Fe_NO_ were inversely associated with FEV_1_ in asthmatic children, suggesting that the inflammatory response of airways most likely influences the decrease in lung function. The effects on inflammatory markers were higher for PM_2.5_ and O_3_ concentrations than for NO_2_. The effect appears on the same day as the exposure and can cumulate over several days, resulting in lung function decrement after 4 or 5 days of cumulative exposure.

Although exposure to outdoor ambient levels of PM_2.5_, NO_2_, and O_3_ has been associated with increased asthma and respiratory symptoms in children, there are few previous longitudinal studies combining inflammatory response and change in lung function in response to air pollution, particularly in nonasthmatic children. Epidemiologic panel studies report that exposure to air pollutants increases the Fe_NO_ levels in susceptible children. Delfino et al. (2006), showed that an IQR increase of 73 μg/m^3^ in 1-hr maximum personal PM_2.5_ was associated with a 0.60-ppb increase in Fe_NO_ (95% CI, 0.14–1.05) in asthmatic children who live in an urban area. A panel study of asthmatic children in Seattle, Washington, reported that ambient PM_2.5_ concentrations were also associated with increased levels of exhaled NO (Mar et al. 2005). We recently reported an increase in Fe_NO_ in asthmatic children living close to roads (50-m buffer) whereas no effect was observed in nonasthmatic children; but no data on lung function or other inflammatory markers were available (Fischer et al. 2002; Holguin et al. 2007; Steerenberg et al. 1999). The present study provides evidence of the adverse effect of traffic-related pollution not only in asthmatic but also in nonasthmatic children.

The effect of PM_2.5_ on IL-8 was stronger in nonasthmatic than in asthmatic children. This was contrary to our expectation. However this has already been reported in another population, in which exposure to diesel exhaust provoked airway inflammation with airway neutrophilia and an increase of IL-8 in healthy subjects shortly after exposure but did not induce neutrophilic response in asthmatic subjects (Stenfors et al. 2004). A potential explanation is that, in asthmatics, IL-10 might inhibit the synthesis of many inflammatory cytokines including IL-8, which has low baseline levels in asthmatic subjects (Barnes and Lim 1998; Stenfors et al. 2004). However, our data are inconclusive on this point. Our interest in estimating the impact of air pollution on IL-8 response is based on the fact that, though not a key player in asthmatic response, its control through a redox-sensitive promoter makes it a good marker of inflammatory response linked to air pollutants (Ichinose et al. 1997). Oxidative effects can increase secretions of proinflammatory cytokines, chemokines, and adhesion molecules and enhance allergic reaction in the airway (Behndig et al. 2006; Takizawa 2004). In particular, oxidative stress has been shown to up-regulate expression of the gene that encodes IL-8 in a variety of cells (Narayanan et al. 1999), leading to the recruitment of neutrophils to sites of inflammation (Diaz-Sanchez and Riedl 2005; Romieu et al. 2008b). In our study, O_3_ (a very potent oxidant) was associated with strong inflammatory responses in both asthmatic and nonasthmatic children.

One difference between asthmatic and nonasthmatic children is the impact of air pollutant exposure on Fe_NO_. Exhaled NO is a sensitive biomarker of airway inflammation (Dinakar 2004); it has been shown to be increased in asthmatic and atopic subjects (Barnes 1996; Gaston et al. 1994; Stamler et al. 2001; Van Amsterdam 2000) and to be a sensitive marker for the respiratory effects of air pollution (Fischer et al. 2002; Holguin et al. 2007). In our study, asthmatic children had a higher baseline Fe_NO_ level than nonasthmatic children, and nonasthmatic children who were atopic had a higher Fe_NO_ baseline level than those who were not [13.9 ppb (IQR = 33.4) vs. 7.7 ppb (IQR = 8.5)]. A significant increase in Fe_NO_ after exposure to air pollutants was observed among the asthmatic children, though no significant response was observed in the nonasthmatic children. However, when we analyzed non-asthmatic atopic children separately, we observed a significant increase of Fe_NO_ after PM_2.5_ exposure, suggesting that atopic children are more responsive to exposure than nonatopic children.

Another contribution of our study is the evaluation of pH of EBC related to air pollutant exposure. We hypothesized that asthmatic children would have a lower exhaled breath pH at baseline than nonasthmatic children and that exposure to air pollutants would lead to a decrease in the pH of exhaled condensate as a marker of lower airway inflammation. The exhaled breath condensate of asthmatics contains a high concentration of reactive oxygen species (ROS) and reactive nitrogen species (RNS), reflecting changes in the lower respiratory tract during inflammation and affecting local pH. This acidification might contribute to asthma pathophysiology (Hunt et al. 2000). Exposure to air pollutants, particularly O_3_, which has a strong oxidative potential and acts in the lower airway, is likely to result in an increase in ROS and RNS. This would affect the pH of the lower respiratory tract, reflecting local inflammation. We found that the pH of EBC decreased in both nonasthmatic and asthmatic children with air pollutant exposure, and significantly so with O_3_ exposure in asthmatic children. Brunetti et al. (2006) observed that the pH of EBC in patients with acute asthma was lower than that of patients with stable asthma, and the amplitude of the effect we observed among asthmatic children in response to O_3_ (16-ppb increase) was similar to that described by these authors. Our results therefore add a plausible biological mechanism to the association of traffic-related emissions and adverse respiratory health effects.

An important issue is the interrelation between inflammatory response and change in lung function in relation to air pollution exposure. Among asthmatic children, FEV_1_ was inversely related to Fe_NO_, IL-8, and PM_2.5_ exposure, but the coefficient of PM_2.5_ decreased when the inflammatory markers were introduced in the models. In contrast, among nonasthmatic children this adjustment strengthened the inverse association between FEV_1_ and PM_2.5._ This suggests that, in asthmatic children, the inflammatory response may partly explain the effect of PM_2.5_ on FEV_1_. We also observed a significant inverse association between decrement in FEV_1_ and increase in Fe_NO_ in asthmatic children. However, given the complexity of inflammatory responses to air pollutants, these results need to be interpreted with caution.

Some limitations must be taken into account when interpreting the results of this study. Daily variations in air pollutant exposure were evaluated through the daily records of the fixed central monitoring locations (RAMA). The temporal variations in each child’s exposure were assumed to follow those at the central monitoring site. To strengthen the validity of this assumption, each child was assigned to the monitoring site closest to his or her home by means of a spatial GIS, providing greater variability in the data. We also monitored PM_2.5_, NO_2_, and O_3_ at the schools and correlated the data from the two sets of monitors. The correlations between the fixed central monitors and the local monitors were 0.77, 0.21, and 0.60 respectively. The lowest correlation was for NO_2_, as in other studies (Kodama et al. 2002), whereas the highest was for PM_2.5_, which might explain why significant changes in lung function were observed only for this pollutant, possibly because of better estimation of exposure. However, specific components of PM_2.5_ also appear to play a role in the adverse effect of this pollutant. When we analyzed data on elemental carbon levels measured in a subsample of PM_2.5_ filters (*n* = 37) from the school-based monitors, we observed that elemental carbon levels were inversely associated with FEV_1_ and FVC in a subgroup of 30 asthmatic children (data not shown) (*p* = 0.005), highlighting the role of heavy vehicle (diesel) emissions as a source of exposure (Janssen et al. 2001) and strengthening the causal relationship with PM_2.5_. Finally, although some degree of exposure may be confounded by other unmeasured factors such as variations in socioeconomic status, this is unlikely in the present study because all our participants came from the same study area and attended the same public school system; and further adjustment by socioeconomic status did not modify our results.

Most of the asthmatic children in this study were classified as having mild intermittent asthma (55%) and only 17.5% as having moderate persistent asthma. This limited our ability to study the impact of air pollution on asthma severity. In previous studies conducted in Mexico City, we had observed that children with moderate to severe asthma were more susceptible to air pollution (Romieu et al. 2004). Our results might therefore underestimate the impact of air pollution on children with more severe asthma.

## Conclusion

Our data show that Fe_NO_ levels, IL-8 levels in nasal lavage, pH of EBC, and changes in lung function are associated with acute exposure to traffic-related air pollutants. These adverse effects were observed in a longitudinal setting in a free-living population, more specifically in a cohort of schoolchildren including nonasthmatic children. Among the asthmatic children, air pollution exposure was related to an increase in Fe_NO_ levels and a decrease in lung function, whereas neutrophil airway inflammation as well as a decrease in lung function was observed in the nonasthmatic children. These results could have significant public health policy implications, because a large proportion of schools in Mexico City and other countries are located very close to roads with heavy traffic.

## Figures and Tables

**Table 1:** Basic characteristics and main outcomes of the study population.

Variable	Asthmatic (*n* = 158)	Nonasthmatic (*n* = 50)	*p*-Value
Sex (% male)^a^	61.9	40.0	0.005
Age (years)^b^	9.6 (7.9–11.0)	9.3 (7.9–11.5)	0.986
Weight (kg)^b^	36.0 (27.0–46.0)	32.0 (26.0–45.0)	0.307
Height (cm)^b^	137.0 (124.5–147.0)	134.0 (127.0–147.0)	0.842
Maternal schooling [years (mean ± SD)]^a^	9.8 ± 3.0	9.3 ± 3.0	0.279
Paternal smoking at home (%)^a^	54.8	45.0	0.424
Maternal smoking at home (%)^a^	41.1	28.6	0.281
Pets at home (%)^a^	56.6	72.7	0.035
Carpet at home (%)^a^	14.4	34.6	0.001
Humidity at home (%)^a^	42.5	41.5	0.899
Prick test positivity (%)	89.0	72.0	0.129
Moderate persistent asthma (%)	17.5		
Mild persistent asthma (%)	26.9		
Mild intermittent asthma (%)	55.0		
Exhaled NO levels (ppb)^b^	23.2 (11.2–46.7)	11.2 (6.0–20.1)	0.000
IL-8 levels (pg/mL) in nasal lavage^b^	157.2 (78.2–295.1)	202.2 (113.0–333.6)	0.002
pH of EBC^b^	7.43 (7.1–7.6)	7.56 (7.3–7.8)	0.632
FEV_1_ [L/sec (mean ± SD)]	1.89 ± 0.66	1.95 ± 0.59	0.595
FVC [L/sec (mean ± SD)]	2.30 ± 0.79	2.25 ± 0.68	0.678
FEV_25–75_ (mean ± SD)	1.89 ± 0.89	2.15 ± 0.88	0.718

Q, quartile.

a Chi-square test.

b Mann-Whitney test [median (Q25–Q75)].

**Table 2:** Air pollutants and climatic variables during the study period.

Variable	Mean ± SD	IQR^a^	Min–max
O_3_ (ppb)			
8-hr moving average	31.6 ± 11.5	22.0	4.9–86.3
NO_2_ (ppb)			
8-hr moving average	37.4 ± 10.9	13.4	14.9–77.6
PM_2.5_ (μg/m^3^)			
8-hr moving average	28.9 ± 2.8	17.5	4.2–102.8
Temperature			
1 hr minimum (°C)	11.4 ± 3.0	4.0	0.7–17.9
Humidity			
1 hr minimum	34.8 ± 11.6	18.1	5.9–70.5

Abbreviations: Max, maximum; Min, minimum.

a IQR (Q25–Q75).

**Table 3:** Association [coefficients per increase in IQR (95% CI)] between exhaled NO, IL-8, pH of EBC, and lung function and air pollutants in asthmatic children living in Mexico City, 2003–2005.

Variable	PM_2.5_ (μg/m^3^)	NO_2_ (ppb)	O_3_ (ppb)
Fe_NO_^a^ (ppb)	1.08 (1.01 to 1.16)*	1.05 (0.98 to 1.12)	1.06 (1.02 to 1.09)*
IL-8^a^ (pg/mL)	1.08 (0.98 to 1.19)	1.03 (0.94 to 1.12)	1.18 (1.04 to 1.34)*
pH_EBC^a^	−0.03 (−0.09 to 0.03)	−0.02 (−0.06 to 0.04)	−0.07 (−0.15 to −0.01)*
FEV_1_^b^ (mL)	−16.0 (−31.0 to −0.13)*	−0.04 (−8.86 to 8.79)	−1.64 (−28.0 to 25.1)
FVC^b^ (mL)	−23.0 (−42.0 to −5.21)*	−1.11 (−12.0 to 9.80)	−13.5 (−45.0 to 19.0)
FEV_25–75_^b^ (mL)	−11.0 (−42.0 to 20.3)	−5.04 (−22.6 to 12.5)	24.3 (−29.0 to 78.2)

Coefficient was calculated for an IQR of pollutants: 17.5 μg/m^3^ for PM_2.5_ , 13.4 ppb for NO_2_, and 22 ppb for O_3_. Lung function models: *n* = 158 and 1,503 measurements. Inflammatory marker models: *n* = 126 and 702 Fe_NO_ measurements, *n* = 119 and 759 IL-8 measurements and *n* = 119 and 551 measurements of pH in EBC.

a Same day exposure: 8-hr moving averages for PM_2.5_ (μg/m^3^), NO_2_ (ppb), and O_3_ (ppb). Inflammatory marker models were adjusted for sex, body mass index, previous day minimum temperature, corticoid use, and chronological time.

b Five-day accumulated average (maximum) PM_2.5_ (μg/m^3^), 4-day accumulated average (maximum) NO_2_ (ppb), and 5-day accumulated moving average O_3_ (ppb). Lung function models were adjusted for sex, body mass index, previous day minimum temperature and chronological time.

* *p* < 0.05.

**Table 4:** Association [coefficients per increase in IQR (95% CI)] between exhaled NO, IL-8, pH of EBC, and lung function and air pollutants in nonasthmatic children living in Mexico City, 2003–2005.

Variable^a^	PM_2.5_ (μg/m^3^)	NO_2_ (ppb)	O_3_ (ppb)
Fe_NO_^a^ (ppb)	0.89 (0.78 to 1.01)*	1.10 (0.99 to 1.23)*	1.11 (0.92 to 1.33)*
IL-8^a^ (pg/mL)	1.16 (1.00 to 1.36)**	1.15 (1.01 to 1.32)**	1.19 (1.00 to 1.45)*
pH_EBC^a^	−0.05 (−0.14 to 0.04)	0.01 (−0.07 to 0.09)	−0.07 (−0.20 to 0.05)
FEV_1_^b^ (mL)	−21.0 (−42.3 to 0.38)*	−6.73 (−22.0 to 8.52)	−21.3 (−66.5 to 23.9)
FVC^b^ (mL)	−29.0 (−52.8 to −4.35)**	−9.51 (−27.0 to 7.97)	−23.6 (−75.0 to 28.1)
FEV_25 –75_^b^ (mL)	−20.0 (−69.0 to 29.0)	−12.1 (−47.0 to 22.7)	−14.5 (−118.7 to 89.5)

Coefficient was calculated for an IQR of pollutants: 17.5 μg/m^3^ for, PM_2.5_, 13.4 ppb for NO_2_, and 22 ppb for O_3_. Lung function models: *n* = 50 and 591 measurements. Inflammatory marker models: *n* = 50 and 302 Fe_NO_ measurements, *n* = 45 and 285 IL-8 measurements and *n* = 44 and 201 measurements of pH in EBC.

a Same day exposure: 8-hr moving averages for PM_2.5_ (μg/m^3^), NO_2_ (ppb), and O_3_ (ppb). Inflammatory marker models were adjusted for sex, body mass index, previous day minimum temperature and chronological time.

b Five-day accumulated average (maximum) PM_2.5_ (μg/m^3^), 4-day accumulated average (maximum) NO_2_ (ppb), and 5-day accumulated moving average O_3_ (ppb). Lung function models were adjusted for sex, body mass index, previous day minimum temperature and chronological time.

* *p* > 0.05, < 0.08.

** *p* < 0.05.
